# Tumor microenvironment-related gene selenium-binding protein 1 (SELENBP1) is associated with immunotherapy efficacy and survival in colorectal cancer

**DOI:** 10.1186/s12876-022-02532-2

**Published:** 2022-10-17

**Authors:** Cheng Zhu, Siya Wang, Yishan Du, Ying Dai, Qian Huai, Xiaolei Li, Yingying Du, Hanren Dai, Wenkang Yuan, Shi Yin, Hua Wang

**Affiliations:** 1 Department of Oncology, the First Affiliated Hospital of Anhui Medical University, 218 Jixi Road, Hefei, 230036 China; 2 Department of Geriatrics, Affiliated Provincial Hospital of Anhui Medical University, Anhui Medical University, Hefei, 230001 China; 3 Department of General Surgery, the First Affiliated Hospital of Anhui Medical University, Hefei, 230036 China

**Keywords:** Colorectal cancer, SELENBP1, Prognosis, Immune infiltration, Immunotherapy, Chemotherapy

## Abstract

**Background:**

Selenium-binding protein 1 (SELENBP1), a member of the selenium-containing protein family, plays an important role in malignant tumorigenesis and progression. However, it is currently lacking research about relationship between SELENBP1 and immunotherapy in colorectal cancer (CRC).

**Methods:**

We first analyzed the expression levels of SELENBP1 based on the Cancer Genome Atlas (TCGA), Oncomine andUALCAN. Chisq.test, Fisher.test, Wilcoxon-Mann-Whitney test and logistic regression were used to analyze the relationship of clinical characteristics with SELENBP1 expression. Then Gene ontology/ Kyoto encyclopedia of genes and genomes (GO/KEGG), Gene set enrichment analysis (GSEA) enrichment analysis to clarify bio-processes and signaling pathways. The cBioPortal was used to perform analysis of mutation sites, types, etc. of SELENBP1. In addition, the correlation of SELENBP1 gene with tumor immune infiltration and prognosis was analyzed using ssGSEA, ESTIMATE, tumor immune dysfunction and rejection (TIDE) algorithm and Kaplan-Meier (KM) Plotter database. Quantitative real-time PCR (qRT-PCR) and western blotting (WB) were used to validate the expression of SELENBP1 in CRC samples and matched normal tissues. Immunohistochemistry (IHC) was further performed to detect the expression of SELENBP1 in CRC samples and matched normal tissues.

**Results:**

We found that SELENBP1 expression was lower in CRC compared to normal colorectal tissue and was associated with poor prognosis. The aggressiveness of CRC increased with decreased SELENBP1 expression. Enrichment analysis showed that the SELENBP1 gene was significantly enriched in several pathways, such as programmed death 1 (PD-1) signaling, signaling by interleukins, TCR signaling, collagen degradation, costimulation by the CD28 family. Decreased expression of SELENBP1 was associated with DNA methylation and mutation. Immune infiltration analysis identified that SELENBP1 expression was closely related to various immune cells and immune chemokines/receptors. With increasing SELENBP1 expression, immune and stromal components in the tumor microenvironment were significantly decreased. SELENBP1 expression in CRC patients affects patient prognosis by influencing tumor immune infiltration. Beside this, SELENBP1 expression is closely related to the sensitivity of chemotherapy and immunotherapy.

**Conclusions:**

Survival analysis as well as enrichment and immunoassay results suggest that SELENBP1 can be considered as a promising prognostic biomarker for CRC. SELENBP1 expression is closely associated with immune infiltration and immunotherapy. Collectively, our study provided useful information on the oncogenic role of SELENBP1, contributing to further exploring the underlying mechanisms.

**Supplementary Information:**

The online version contains supplementary material available at 10.1186/s12876-022-02532-2.

## Introduction

CRC ranks third in incidence and second in mortality among all malignancies, and with environmental changes and an aging population, the incidence and mortality rates of CRC continue to rise significantly [1]. Currently, clinical treatment for CRC is still based on surgery, radiotherapy and targeted drugs, but the overall cancer treatment was shown limited efficacy. In recent years, the rapid development of immunotherapy in the field of oncology has provided new therapeutic strategies for CRC, and immunotherapy of CRC has become a hot spot for research. Therefore, it is particularly important to find biological markers for prognosis associated with immune infiltration in CRC patients.

Selenium is the only essential trace element that is genetically regulated and can be converted in vivo into selenium-containing amino acids for insertion into various proteins, regulating the activity of different selenoproteins [2]. Previous studies have shown that selenium and selenium-binding proteins play an important role in the regulation of cancer immunity. Numerous clinical studies support the benefit of selenium supplementation on the immune system during cancer treatment [3]. In patients with leukemia/lymphoma and neutropenia treated with selenium supplementation, the patients showed a significant increase in neutrophil count and an enhanced immune response [4, 5]. Studies in animal models of melanoma or breast cancer have found that higher selenium intake reduces tumor growth and also has immune-enhancing effects. When its immune response is analyzed, the main effect is to enhance Th1 immunity and reduce regulatory T cells (Treg) and myeloid-derived suppressor cells that suppress anti-tumor immunity [6–8]. In addition, research targeting selenium nanoparticles and tumor prevention and treatment has become a new strategy to modulate the immune microenvironment of tumors. Song et al. found that the transformable selenium nanodrug (SeNP@LNT) could change the "cold" state of malignant pleural effusion to a "hot" state. On the one hand, it improves the suppressed state of immune cells by regulating and activating various immune cells in malignant pleural effusion; on the other hand, it optimizes the tumor immune microenvironment by promoting the production of metabolites related to tumor growth inhibition and immune response activation in the microenvironment [9]. In the case of successive immune cell therapy, selenium nanoparticles effectively enhanced the anti-tumor effect of immune cells (CIK cells). The mechanism of action of selenium nanoparticles is that it is mainly metabolized to SeCys (selenocysteine) and SeIV in tumor cells and immune cells, and selenocysteine is an important active center of intracellular selenoprotein. It was further detected that the addition of selenium nanoparticles significantly up-regulated the expression of various selenoproteins such as SELK, SELO, SELP, SelR, SELES, SELT, SELW, Gpx2, TrxR1 and Sep15 in cells, thus enhancing the anti-tumor effect of immune cell therapy [10]. Undoubtedly, selenoprotein works through the immune system has been an extremely important part of its fight against cancer. Previous studies have only focused on the significant decrease or even disappearance of SELENBP1 expression levels in most cancer tissues like prostate, ovarian, lung, and colorectal cancers, and closely correlated with tumor prognosis, but the mechanisms have not been explored [11–14]. Considering the effects of selenium and other members of the selenoprotein family on the immune status of tumors, we hypothesized that SELENBP1 may affect the prognosis of CRC by influencing its immune infiltration.

We evaluated the association of SELENBP1 gene expression with immune infiltration and prognosis in CRC by analyzing clinical indicators and survival data from several databases. Our study revealed the molecular mechanism of SELENBP1 dysregulation in CRC, and the potential role in its development. In addition, we demonstrated the association between SELENBP1 and immunotherapy and chemotherapy. This may provide new insights into the treatment of CRC.

## Methods

### TCGA/GTEx

TCGA (http://www.cancergenome.nih.gov/) is a very important cancer database that containing clinical data, genomic variants, m/mi/lncRNA expression, DNA methylation and other data on various human cancers. The Genotype-tissue expression (GTEx) (https://cancergenome.nih.gov/) database is a normal tissue gene expression database, which is often used in conjunction with TCGA. We downloaded all SELENBP1 clinicopathological data, mRNA expression, methylation and other data from TCGA website in CRC, and collected transcripts per kilobase of exon model per million mapped reads (TPM) expression values of SELENBP1 in normal colorectal tissues from GTEx database to add supplementary control tissues.

### Oncomine database

The Oncomine database (https://www.oncomine.org/resource/main.html) contains comprehensive cancer mutation profiles, gene expression data and relevant clinical information to facilitate the discovery of new biomarkers [15]. Based on Oncomine platform, the expression levels of SELENBP1 mRNA in various subtypes of CRC and normal control tissues were analyzed.

### UALCAN cancer database

The UALCAN ( http://ualcan.path.uab.edu/index.html) database is an interactive web-based resource platform that facilitate gene queries of the TCGA database, analyze and identify tumor-associated biomolecular markers, and analyze the molecular mechanisms of tumorigenesis, progression and metastasis, which are important for the diagnosis, treatment and prognosis of tumors, and can make researchers easier access to TCGA data and analysis results [16]. This study used the UALCAN database to analyze the protein expression of SELENBP1 in CRC tissues.

### CancerSEA database

CancerSEA (http://biocc.hrbmu.edu.cn/CancerSEA/) is a single-cell sequencing database. It decodes the different functions of cancer cells from single-cell level, thus revealing the functional heterogeneity of cancer cells [17]. We analyzed the relationship between SELENBP1 expression and the 14 functional states of cancer cells using CancerSEA.

### Gene set enrichment analysis

To investigate the potential mechanisms of SELENBP1 in CRC, we analyzed TCGA transcriptome data and divided colorectal cancer samples into high and low SELENBP1 expression groups. Differentially expressed genes (DEGs) were screened between the two groups, and GO/KEGG analysis was performed on the upregulated DEGs using the clusterProfiler R package [18–21]. In addition, we performed GSEA analysis of the differential genes of SELENBP1, we analyzed the "c2.cp.v7.2.symbols.gmt" gene set in the MSigDB Collections genome database using the clusterProfiler package, and the number of calculations 1000 times was chosen [22, 23]. Results with normalized enrichment score (NES) < − 1.5, P.adjust < 0.05 and false discovery rate (FDR) q < 0.25were considered to be significantly enriched.

### cBioPortal database

The cBioPortal database (http://www.cbioportal.org/index.do) contains data from 200 tumor genomic studies, including large tumor research projects such as TCGA and International Cancer Genome Consortium (ICGC). It can be used to analyze the mutation of a gene in different cancers, including the mutation site, type, amino acid change and the corresponding protein 3D structure, etc. [24]. We used the cBioPortal database to study the expression and mutation of SELENBP1, etc.

### Immune infiltration analysis

The association of SELENBP1 and 24 immune cells was analyzed by the ssGSEA algorithm in the "GSVA" package [25, 26]. We used ESTIMATE algorithm to analyze the relationship between SELENBP1 expression and ImmuneScore, StromalScore and ESTIMATEScore [27]. Then, we analyzed the correlation of SELENBP1 expression with immune checkpoint gene levels, immune chemokines/receptors, tumor mutation burden (TMB) and microsatellite instability (MSI) using the Spearman correlation analysis. The scores of the four different immunophenotypes (antigen-presenting, effector, suppressor, and checkpoint) were calculated separately by the immunophenoscore (IPS), and the IPS z-score was the integration of the four, and the higher the IPS z-score, the more immunogenic the sample [28]. In addition, we performed a prognostic analysis of SELENBP1 expression levels in different immune cell subsets using the Kaplan⁃Meier Plotter database ( http://kmplot.com/analysis/) [29].

### Drug sensitivity analysis

The Genomics of Cancer Drug Sensitivity (GDSC) database (https://www.cancerrxgene.org/), which contains a large number of drug sensitivity and genomic datasets [30]. The Cancer Therapeutics Response Portal (CTRP) database (http://www.broadinstitute.org/ctrp/) covers associations between compound sensitivities and genetic or genealogical characteristics for a wide range of cancer cell lines [31–33]. The tumor immune dysfunction and rejection (TIDE) algorithm is a more accurate predictor of the efficacy of immune checkpoint blockade therapy (ICB) [34]. We analyzed the sensitivity of SELENBP1 expression to chemotherapeutic agents using the GDSC, CTRP database and the efficacy of ICB treatment using the TIDE algorithm.

### Western blotting and RT-PCR assay

Tissue protein was extracted by RIPA lysis-buffer containing with protease inhibitor and phosphatase inhibitors, and protein content was measured by the BCA assay (Beyotime, Shanghai, China). Tissue protein (20 μg) were separated via 8% SDS-PAGE. After the run in electrode buffer the gel was transferred to PVDF membranes (Merck Millipore, MA, USA). Membranes were blocked with 5% bovine serum albumin in TBST (TBS with 0.1% Tween-20), and incubated with primary antibodies specific for SELENBP1 (1:1000, Proteintech) and β-actin (1:10,000, Proteintech) overnight at 4℃. PVDF membranes were washed three times for 5 min in TBST and incubated with anti-rabbit secondary antibody (1:10,000, Proteintech) or anti-mouse secondary antibody (1:10,000, Proteintech) for 1 h at room temperature. The membranes were washed as described above and developed using enhanced chemiluminescence assay (PJ2022-09-57).

Paracancer tissues and cancer tissues of total RNA were collected and isolated using TRIzol (Vazyme, Nanjing, China). Before synthesizing cDNA, gDNA-wiper mix (Vazyme, Nanjing, China) was used to remove genomic DNA*,* and the total RNA was reverse transcribed with HiScript QRT SuperMix for qPCR Kit (Vazyme, Nanjing, China). The cDNA was amplified using SYBR Green Master mix (Vazyme, Nanjing, China). The amount of target gene was calculated by 2-ΔΔCt with as reference gene β-actin. The PCR primers used were as follows: SELENBP1 forward: ACCCAGGGAAGAGATCGTCTA, reverse: ACTTGGGGTCAACATCCACAG; β-actin forward: CATGTACGTTGCTATCCAGGC, reverse: CTCCTTAATGTCACGCACGAT.

#### Immunohistochemical staining and patient information

Section of colorectal cancer tissue were obtained from 5 operable patients who underwent curative surgery in The First Affiliated Hospital of Anhui Medical University. The study was approved by the ethics committee of First Affiliated Hospital of Anhui Medical University (PJ2022-09-57).

Tumor tissues were used by immunohistochemical staining, performed on formalin-fixed paraffin-embedded, and cut into 4-μm thickness section. After overnight incubation with anti-SELEBNP1 antibody (1:200, Proteintech), the sections were incubated with secondary antibody, visualized with DAB detection.

### Statistical analysis

GraphPad Prism and R software were used for data analysis and visualization. RT-PCR results were compared using Students' t-test. Chisq.test, Fisher.test, Wilcoxon rank sum test and Logistic regression were used to correlate SELENBP1 expression and clinicopathological characteristics. The relationship between SELENBP1 gene expression and survival of patients was analyzed using the Kaplan–Meier (KM) model. Correlations between the SELENBP1 gene and other genes was performed by using Spearman's correlation analysis. P < 0.05 indicates a significant difference.

## Results

### The expression of SELENBP1 is reduced in CRC patients

**Fig. 1:**
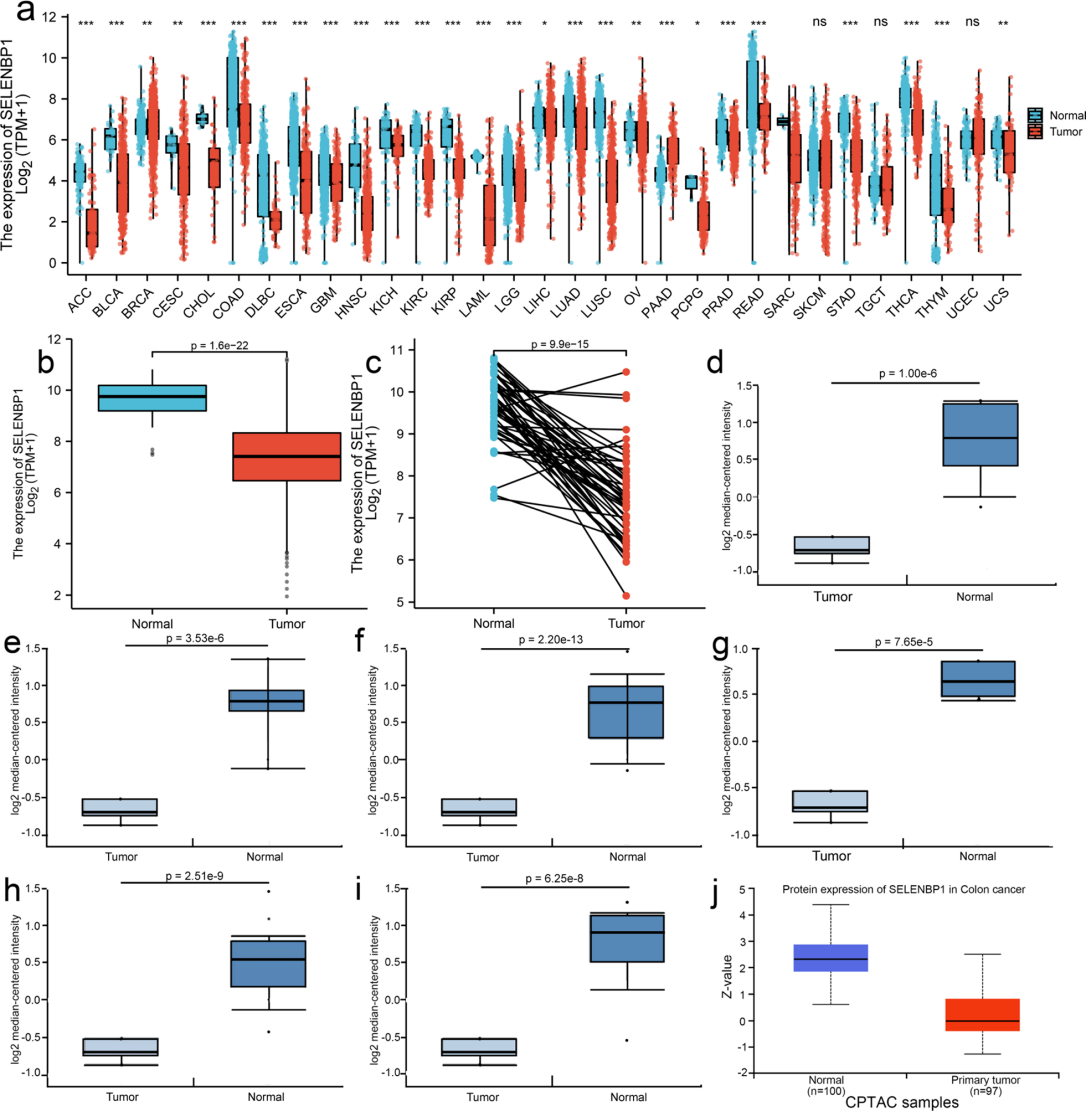
Pan-cancer expression analysis of SELENBP1 and its specific expression in CRC. **a** Pan-cancer SELENBP1 expression analysis. **b** Expression of SELENBP1 in unpaired samples of CRC and normal tissue in the TGCA database. **c** SELENBP1 expression in 50 pairs of CRC and non‐cancerous adjacent tissues from TGCA datasets. **d–i** The mRNA expression of SELENBP1 in different subtypes of CRC in the Oncomine database, in order, they were Rectosigmoid adenocarcinoma **(d)**, Rectal adenocarcinoma **(e)**, Colon adenocarcinoma **(f)**, Rectal mucinous adenocarcinoma **(g)**, cecum adenocarcinoma **(h)**, Colon mucinous adenocarcinoma **(i)**. **j** The protein expression of SELENBP1 in CRC and normal tissues analyzed by UALCAN cancer database. *, *p* < 0.05; **, *p* < 0.01; ***, *p* < 0.001

We first assessed SELENBP1 expression in pan-cancer data from TCGA and GTEx. The analysis revealed SELENBP1 expression was decreased in 27 different types of tumors, including ACC, BLCA, BRCA, CESC, CHOL, COAD, DLBC, ESCA, HNSC, GBM, KICH, KIRC, KIRP, LAML, LGG, LIHC, LUAD, LUSC, OV, PAAD, PCPG, PRAD, STAD, READ, THCA, THYM, and UCS. In contrast, its expression of SELENBP1 was high in SKCM, TGCT, and UCEC (Fig. 1a). Recent studies have found that selenoproteins play an important role in the intestinal immune system. Huang et al. characterized the colonic immune cell profiles and metabolic microenvironment of patients with primary untreated IBD by single-cell transcriptome sequencing and metabolome resolution, and found disease-specific immune cell subpopulations and metabolite alterations in CD or UC. Functional screening of metabolites by in vitro experiments revealed that specifically reduced selenium-containing metabolites could regulate the differentiation of a unique group of Th1-like cells in CD. Further in vitro and in vivo studies confirmed that selenium supplementation can effectively inhibit Th1 differentiation and promote the relief of intestinal inflammation [35]. Based on this we focused on the role of SELENBP1 in colorectal cancer to investigate. The gene expression level of SELENBP1 was significantly lower in tumors than normal colorectum tissues (Fig. 1b). Meanwhile, we also performed an analysis of SELENBP1 expression in 50 tumor samples and their matched adjacent tissues. The results showed that CRC tissues lower expressed SELENBP1 (Fig. 1c). Then, we used the Oncomine database to analyze the expression of SELENBP1 in each subtype of CRC. It was shown that SELENBP1 expression was reduced in rectosigmoid adenocarcinoma, rectal adenocarcinoma, colon adenocarcinoma, rectal mucinous adenocarcinoma, cecum adenocarcinoma, and colon mucinous adenocarcinoma, compared with the normal tissues (Fig. 1d–i and Table 1). Based on the Ualcan database, we found that SELENBP1 protein expression was also decreased in colon cancer (Fig. 1j).

**Table 1:** SELENBP1 expression in different subtypes of CRC and normal tissues using the Oncomine database

Colorectal cancer subtype	*P* value	t test	Fold change	Patient number	Reference
Cecum adenocarcinoma	2.51E−9	9.755	2.211	17	PMID: 17615082
Rectal mucinous adenocarcinoma	7.65E−5	11.354	2.585	4	PMID: 17615082
Rectosigmoid adenocarcinoma	1.00E−6	8.918	2.681	10	PMID: 17615082
Rectal adenocarcinoma	3.53E−6	9.326	2.748	8	PMID: 17615082
Colon mucinous adenocarcinoma	6.25E−8	9.366	2.719	13	PMID: 17615082
Colon adenocarcinoma	2.20E−13	14.956	2.576	41	PMID: 17615082

### Q-PCR and WB demonstrate low expression of SELENBP1 in CRC

**Fig. 2:**
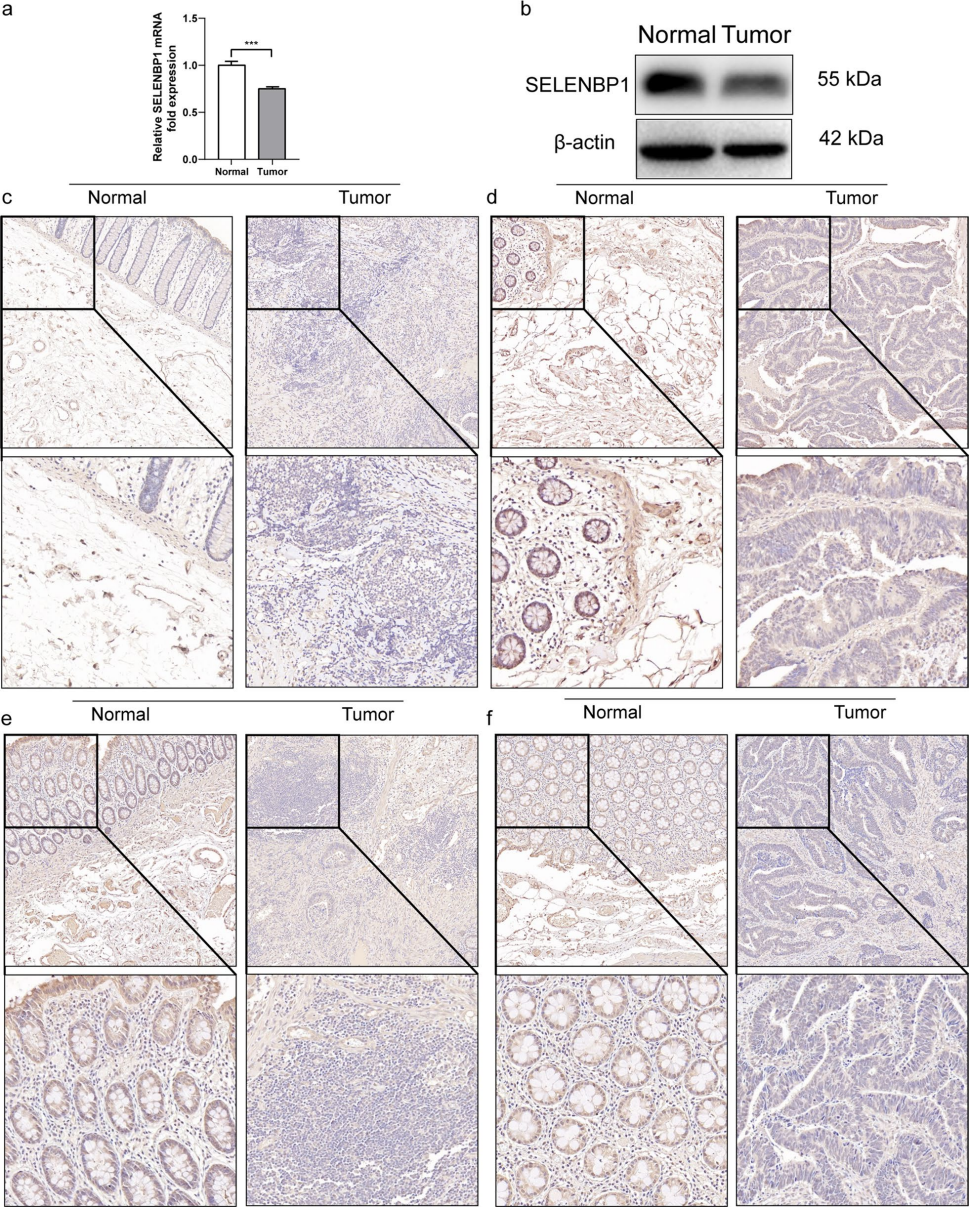
SELEBNP1 expression was lower in CRC tissue than matched normal tissue. **a–b** mRNA and protein expression level of SELEBNP1 were measured by qPCR and western blotting. β-action was regarded as a loading control in colorectal cancer tissues and matched normal tissues. **c–f** SELEBNP1 expression were detected by immunohistochemistry staining in patients

Next, we further confirmed the expression of SELENBP1 in CRC by q-PCR, WB and IHC. As shown in Fig. 2a, SELENBP1 mRNA was reduced in CRC compared with non-cancerous tissues. The results of IHC and WB showed that the protein level of SELENBP1was significantly lower in tumor tissues than their matched adjacent tissues (Fig. 2b–f, Additional file 1: Fig. S1).

### Relationship between SELENBP1 expression and clinical parameters in CRC tissues

To understand whether there were differences in SELENBP1 expression in different clinical indices, we performed statistical analysis by Chisq.test, Fisher.test, Wilcoxon-Mann–Whitney test and logistic regression. The results showed no significant differences between the expression levels of SELENBP1 and clinical characteristics (Table 2, Table 3).

**Table 2:** Relationship between SELENBP1 expression and clinical characteristics

Characteristic	Low expression of SELENBP1	High expression of SELENBP1	*P*
n	309	310	
T stage, n (%)			0.088
T1	5 (1.6%)	15 (4.9%)	
T2	49 (15.9%)	56 (18.1%)	
T3	215 (69.8%)	207 (67%)	
T4	39 (12.7%)	31 (10%)	
N stage, n (%)			0.563
N0	169 (54.9%)	182 (59.1%)	
N1	78 (25.3%)	72 (23.4%)	
N2	61 (19.8%)	54 (17.5%)	
M stage, n (%)			0.873
M0	230 (83.6%)	229 (84.5%)	
M1	45 (16.4%)	42 (15.5%)	
Pathologic stage, n (%)			0.331
Stage I	44 (14.7%)	61 (20.3%)	
Stage II	119 (39.8%)	108 (36%)	
Stage III	92 (30.8%)	87 (29%)	
Stage IV	44 (14.7%)	44 (14.7%)	
Primary therapy outcome, n (%)			0.126
PD	20 (14.2%)	13 (8.3%)	
SD	4 (2.8%)	1 (0.6%)	
PR	5 (3.5%)	10 (6.4%)	
CR	112 (79.4%)	132 (84.6%)	
Gender, n (%)			0.902
Female	143 (46.3%)	146 (47.1%)	
Male	166 (53.7%)	164 (52.9%)	
Race, n (%)			0.051
Asian	8 (4%)	4 (2.4%)	
Black or African American	27 (13.4%)	38 (22.8%)	
White	167 (82.7%)	125 (74.9%)	
Age, n (%)			0.899
< = 65	133 (43%)	136 (43.9%)	
> 65	176 (57%)	174 (56.1%)	
Weight, n (%)			0.848
< = 90	125 (72.3%)	106 (70.7%)	
> 90	48 (27.7%)	44 (29.3%)	
Height, n (%)			0.952
< 170	78 (47.6%)	68 (48.6%)	
> = 170	86 (52.4%)	72 (51.4%)	
Residual tumor, n (%)			0.567
R0	218 (92.8%)	232 (90.3%)	
R1	2 (0.9%)	4 (1.6%)	
R2	15 (6.4%)	21 (8.2%)	
BMI, n (%)			0.736
< 25	51 (31.1%)	47 (33.6%)	
> = 25	113 (68.9%)	93 (66.4%)	
CEA level, n (%)			0.967
< = 5	118 (63.1%)	134 (63.8%)	
> 5	69 (36.9%)	76 (36.2%)	
Perineural invasion, n (%)			0.967
No	95 (73.6%)	77 (74.8%)	
Yes	34 (26.4%)	26 (25.2%)	
Lymphatic invasion, n (%)			0.515
No	168 (60.9%)	163 (57.8%)	
Yes	108 (39.1%)	119 (42.2%)	
History of colon polyps, n (%)			0.705
No	174 (67.4%)	190 (69.3%)	
Yes	84 (32.6%)	84 (30.7%)	
Colon polyps present, n (%)			0.775
No	110 (70.5%)	97 (68.3%)	
Yes	46 (29.5%)	45 (31.7%)	
Neoplasm type, n (%)			0.286
Colon adenocarcinoma	233 (75.4%)	221 (71.3%)	
Rectum adenocarcinoma	76 (24.6%)	89 (28.7%)

**Table 3:** Logistic regression analysis of the correlation between SELENBP1 expression and clinical characteristics

Characteristics	Total(N)	Odds Ratio (OR)	P value
T stage (T3&T4&T2 vs. T1)	641	0.324 (0.104–0.847)	0.031
N stage (N1&N2 vs. N0)	640	0.950 (0.694–1.300)	0.749
M stage (M1 vs. M0)	564	1.036 (0.658–1.631)	0.879
Pathologic stage (Stage II&Stage III&Stage IV vs. Stage I)	623	0.754 (0.498–1.138)	0.180
Gender (Male vs. Female)	644	1.013 (0.743–1.380)	0.937
Age (> 65 vs. < = 65)	644	0.837 (0.612–1.144)	0.265
Weight (> 90 vs. < = 90)	348	0.935 (0.588–1.482)	0.774
Height (> = 170 vs. < 170)	329	0.908 (0.587–1.403)	0.664
BMI (> = 25 vs. < 25)	329	0.935 (0.589–1.489)	0.778
Residual tumor (R1&R2 vs. R0)	510	1.695 (0.891–3.344)	0.115
CEA level (> 5 vs. < = 5)	415	0.915 (0.614–1.363)	0.661
Perineural invasion (Yes vs. No)	235	0.992 (0.549–1.786)	0.980
Lymphatic invasion (Yes vs. No)	582	1.258 (0.903–1.756)	0.176
History of colon polyps (Yes vs. No)	555	0.883 (0.618–1.261)	0.494
Colon polyps present (Yes vs. No)	323	0.979 (0.608–1.573)	0.930
Neoplasm type (Rectum adenocarcinoma vs. Colon adenocarcinoma)	644	1.341 (0.941–1.915)	0.105

### Low SELENBP1 expression impacts the prognosis of CRC patients

**Fig. 3:**
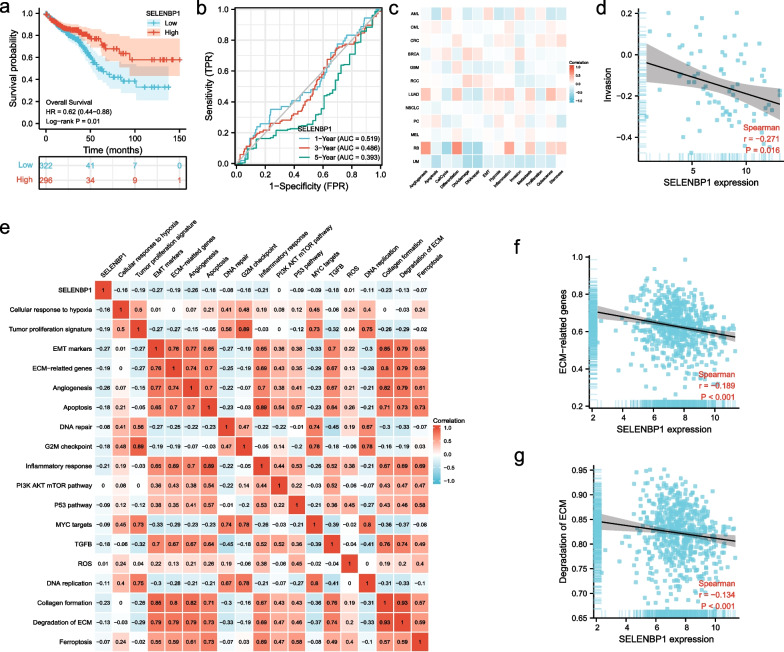
Prognostic analysis of gene signature in the TCGA set. **a** KM survival curve of SELENBP1 expression. **b** SELENBP1 expression predicts OS in CRC patients. **c** Fourteen different functional states of SELENBP1 in nine cancers. **d** Relationship between SELENBP1 expression and invasiveness of CRC. **e** Heat map showing SELENBP1 and GSVA scores of cancer hallmark features. **f** Correlation of SELENBP1 with ECM-relatted genes pathway. **g** Correlation of SELENBP1 with Degradation of ECM pathway

We evaluated the relationship between SELENBP1 expression levels and patient prognosis using the TCGA database. KM survival curves showed that overall survival (OS) was significantly prolonged in the high expression group (Fig. 3a). We also used time-dependent receiver operating characteristic (ROC) curves (Fig. 3b) to compare the predictive accuracy of the SELENBP1 expression, with AUCs of 0.519, 0.486, and 0.393 for 1 year-, 3 year-, and 5 year- OS, respectively, and it demonstrated the good sensitivity of this prognostic model in predicting long-term prognosis of CRC. In addition, we found that OS was also significantly prolonged in patients with high SELENBP1 expression in HNSC, LUAD, LIHC, THCA, MESO, SARC, and BLCA (Additional file 1: Fig. S2). To better understand the potential mechanisms of SELENBP1 in colorectal carcinogenesis, we investigated the relationship between SELENBP1 and 14 functional states of cancer in the CancerSEA database. The results showed that SELENBP1 expression was negatively correlated with CRC invasiveness (Fig. 3c–d). In addition, we also performed GSVA analysis using the TCGA bulk RNA-seq dataset data. The heat map combined with the difference in GSVA scores showed that SELENBP1 expression was negatively correlated with multiple cancer species-related pathways like Tumor proliferation signature, Cellular response to hypoxia, EMT markers, TGFB, etc. (Fig. 3e). We also observed that as SELENBP1 expression increased, extracellular matrix (ECM)-related pathways decreased (Fig. 3f–g). ECM is a fibrin and proteoglycan scaffold capable of maintaining tissue structure and plays a key role in cancer invasion. This result is consistent with the above, and based on these findings, we conclude that SELENBP1 may inhibit the development of CRC.

### Gene set enrichment analysis of SELENBP1

**Fig. 4:**
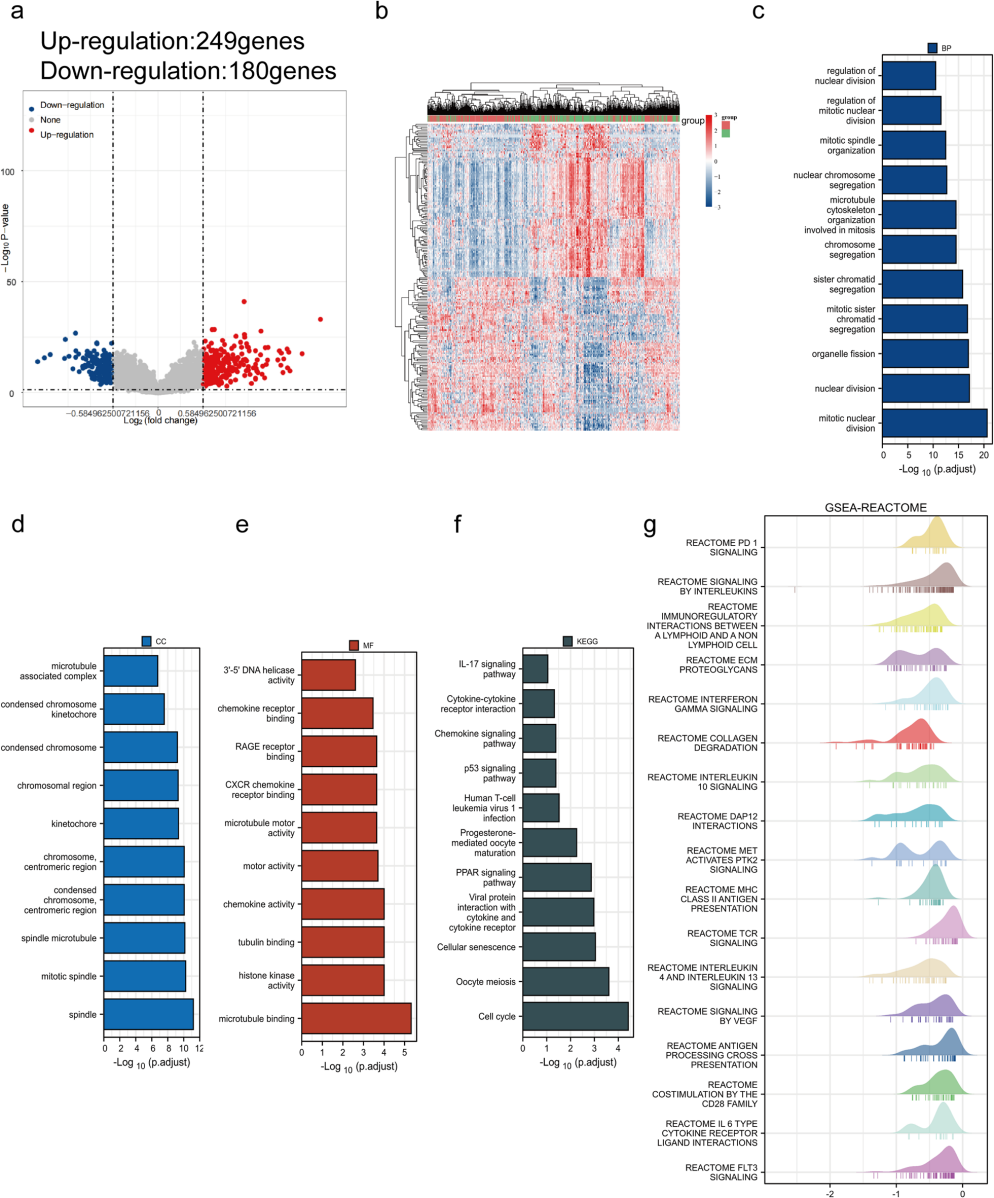
Enrichment analysis of SELENBP1. **a** Volcano plot of DEGs between SELENBP1 high and low expressing CRC samples. **b** Expression heat map of SELENBP1 expression-related DEGs (The 50 most differentially altered up-regulated genes and 50 down-regulated genes). **c–f** GO/KEGG functional enrichment analysis of DEGs with up-regulated SELENBP1 expression. **(g)** GSEA analysis of genes differentially expressed with SELENBP1 (Reactome pathway)

The exact pathway that SELENBP1 may regulate in CRC remains unclear. For this purpose, we analyzed TCGA transcriptome data and divided colorectal cancer samples into high and low SELENBP1 expression groups. Differentially expressed genes (DEGs) were screened between the two groups (|log2FC|> 1.5, adjusted *P* < 0.05). The volcano plot showed that 429 genes were differentially expressed, containing 249 up-regulated and 180 down-regulated genes (Fig. 4a). The heat map shows the corresponding hierarchical clustering analysis of these DEGs. Due to the large number of differential genes, the 50 up-regulated genes and 50 down-regulated genes with the largest differential changes are shown here (Fig. 4b). We performed GO/KEGG analysis on the up-regulated DEGs, and the results of GO analysis showed that these DEGs were enriched in nuclear division, CXCR chemokine receptor binding, chemokine activity, chemokine receptor binding, KEGG analysis showed that these DEGs were enriched in IL-17 signaling pathway, cytokine-cytokine receptor interaction, chemokine signaling pathway, p53 signaling pathway, and human T-cell leukemia virus 1infection were enriched (Fig. 4c–f). In addition, we also performed GSEA analysis for all genes in the differentially expressed list. The analysis revealed PD-1 signaling, signaling by interleukins, TCR signaling, collagen degradation, MHC class II antigen presentation, costimulation by the CD28 family, antigen processing cross presentation and other pathways were significantly enriched (Fig. 4g).

### Gene alteration and methylation analysis of SELENBP1

**Fig. 5:**
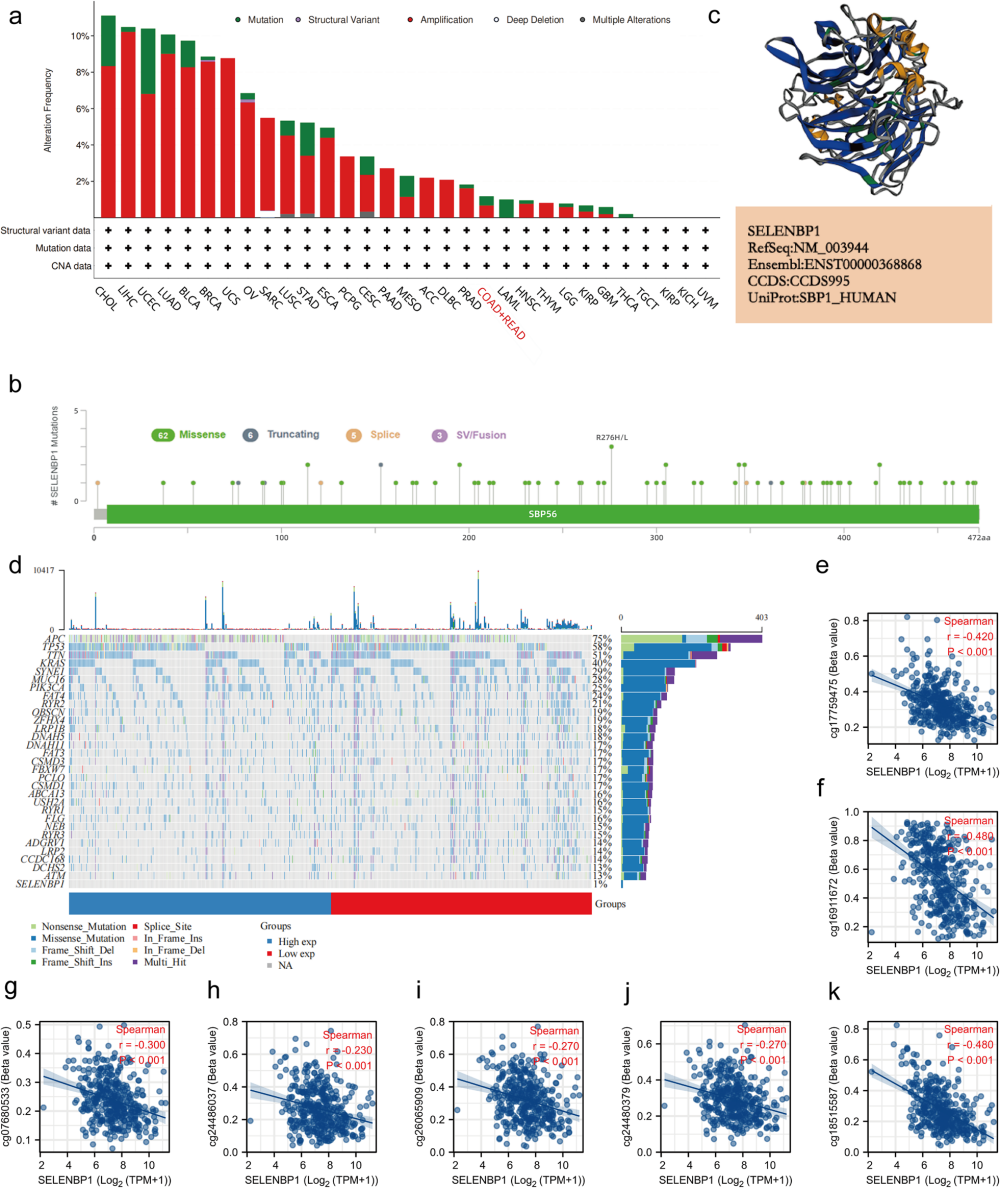
Mutation and methylation characteristics of SELENBP1 in CRC. **a** Mutation types of SELENBP1 and their frequencies. **b** Mutation sites of SELENBP1. **c** 3D structure of SELENBP1 containing mutated sites. **d** Somatic mutations in CRC with high and low expression of SELENBP1. **e–k** Correlation between the degree of methylation at different methylation sites and SELENBP1 expression

We analyzed the alteration status of SELENBP1 gene expression in different tumor samples based on the TCGA database, as shown in Fig. 5a. SELENBP1 is less frequently altered in CRC patients (< 2%), including "mutations" and "amplifications". Figure 5b shows the type, site and corresponding location of SELENBP1 mutations. We further show the 3D structure of the SELENBP1 protein containing the mutation site (Fig. 5c). In order to understand the somatic mutations in CRC patients, we analyzed the mutation data using the "maftools" package of R software [36]. The horizontal histograms showed a high frequency of mutations in CRC patients for APC (75%), TP53 (58%), TTN (51%), KRAS (40%) and a low frequency of mutations in SELENBP1 (1%, Fig. 5d). DNA methylation alters the appearance and structure of DNA. Methylation directly prevents DNA recognition and binding to transcription factors or interferes with the binding of transcription factors by attracting other factors to preferentially bind to DNA, resulting in transcriptional repression or silencing of genes [37]. By analyzing the TCGA database we found that SELENBP1 expression in CRC was associated with DNA methylation sites such as cg17759475 (r = − 0.42, *P* < 0.001), cg16911672 (r = − 0.47, *P* < 0.001), cg07680533 (r = − 0.300, *P* < 0.001) cg24486037 (r = − 0.230, *P* < 0.001), cg26065909 (r = − 0.270, *P* < 0.001), cg24480379 (r = − 0.270, *P* < 0.001), and cg18515587 (r = − 0.480, *P* < 0.001), with a significant negative correlation in methylation (Fig. 5e–k).

### Correlation analysis between SELENBP1 expression and immune infiltration

The immune status of the tumor microenvironment can accurately reflect the body's anti-tumor immune response [38]. Thus, we studied whether SELENBP1 expression was associated with the level of immune infiltration in CRC. We evaluated the correlation of SELENBP1 with 24 immune cell subsets in CRC (Additional file 1: Fig. S3a). Analysis revealed that SELENBP1 positively correlated with eosinophils, B cells and Th17 cells, and negatively correlated with macrophages, Th1 cells, neutrophils, Th2 cells, Tgd, NK cells, T helper cells, Tem, cytotoxic cells, Tcm, CD8 T cells, aDC and DC (Additional file 1: Fig. S3b–r). It was shown that the activity of CRC cells was significantly suppressed in the eosinophilic immune microenvironment [39]. B cells inhibit tumor progression via secretion of cytokines, presentation of antigens, and secretion of antibodies [40]. Macrophages increased the invasion and metastasis of CRC cells, the number of macrophages in tumor was correlated considerably with the depth of tumor invasion, lymph node metastasis, and tumor stages [41]. In addition, increasing neutrophils in the tumor are associated with a malignant phenotype, which could predict a poor prognosis marker in CRC [42]. Through the above analysis, we have found that SELENBP1 mRNA expression levels are associated with immune infiltration in the tumor microenvironment, and that CRC patients with low SELENBP1 mRNA expression levels have shorter OS than those with low SELENBP1 mRNA expression levels. Therefore, it was hypothesized that the prognosis of patients with SELENBP1 mRNA expression level was associated with the degree of immune infiltration. To prove this conclusion, we used the Kaplan ⁃Meier Plotter database to analyze the prognosis of CRC patients based on the expression level of SELENBP1 in the relevant immune cell subgroups, and found that patients with high SELENBP1 mRNA expression level in the CD4+ T cell high-infiltration group, eosinophil high-infiltration group, Th1 cell high-infiltration group and Th2 cell high-infiltration group had longer OS. Whereas, the expression level of SELENBP1 in the immune cell low infiltration group was not related to OS. This suggests that the prognostic impact of SELENBP1 mRNA expression level in CRC patients may be associated with tumor immune infiltration to some extent (Additional file 1: Fig. S4).

**Fig. 6:**
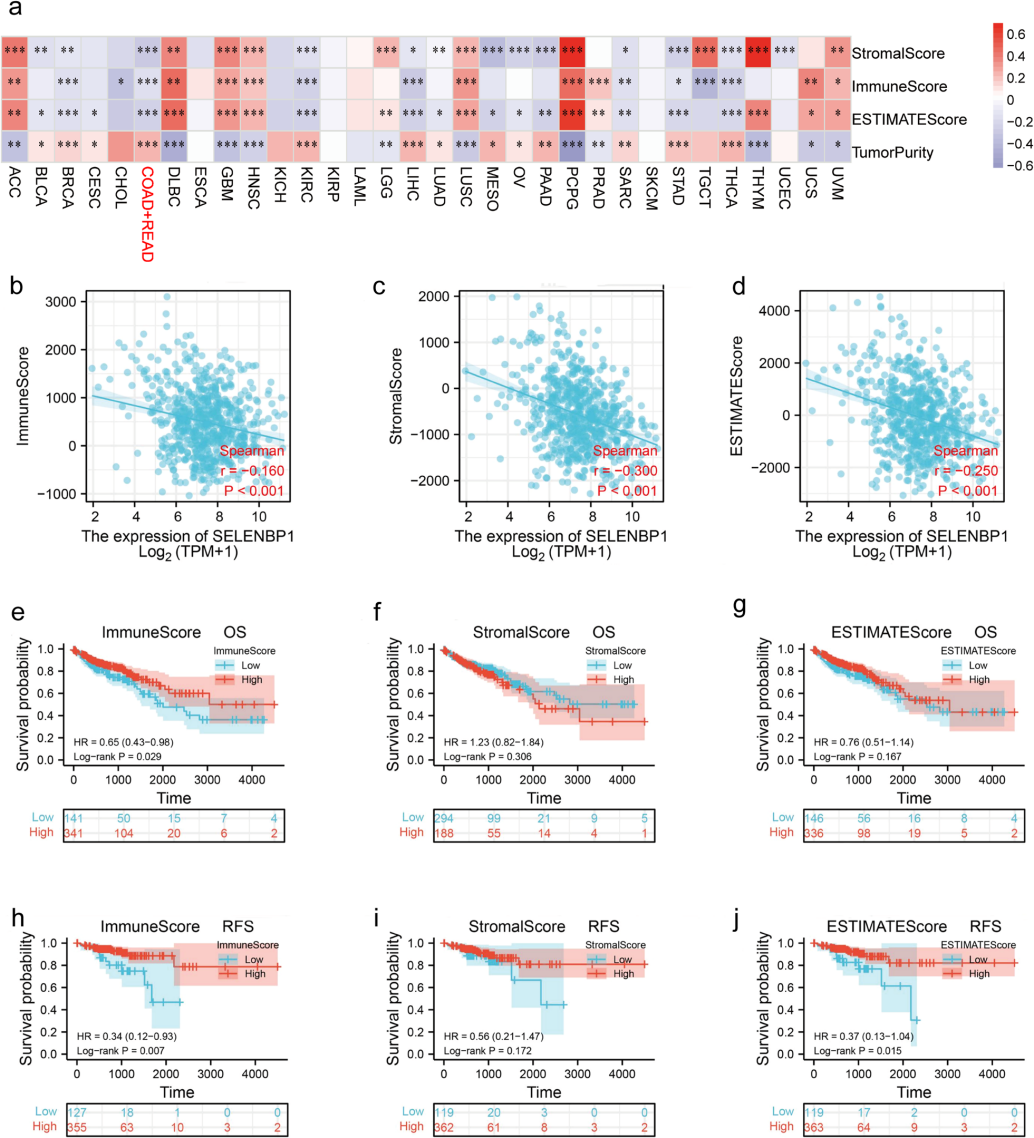
Correlation of SELENBP1 expression with StromalScore, ImmuneScore, ESTIMATEScore in CRC. **a** Heat map showing the correlation of SELENBP1 expression with StromalScore, ImmuneScore, ESTIMATEScore and TumorPurity in different cancer species. **b–d** Scatter plots showing the correlation of StromalScore, ImmuneScore and ESTIMATEScore with SELENBP1 expression in colorectum. **e–j** OS, RFS of CRC patients with different StromalScore, ImmuneScore, ESTIMATEScore

Then we quantify the immune and stromal components in tumors based on the ImmuneScore and StromalScore (Fig. 6a). The results show with the increase of SELENBP1 expression, StromalScore, ImmuneScore and ESTIMATEScore all showed a significant decrease (Fig. 6b–d). We also analyzed the OS, recurrence-free survival (RFS) of patients according to the immune component ratios, stromal component ratios, and the combined ratio of the two components (Fig. 6e–j). The results showed that OS and RFS were significantly prolonged in patients with a high proportion of immune components and RFS was significantly prolonged with high ESTIMATEScore.

**Fig. 7:**
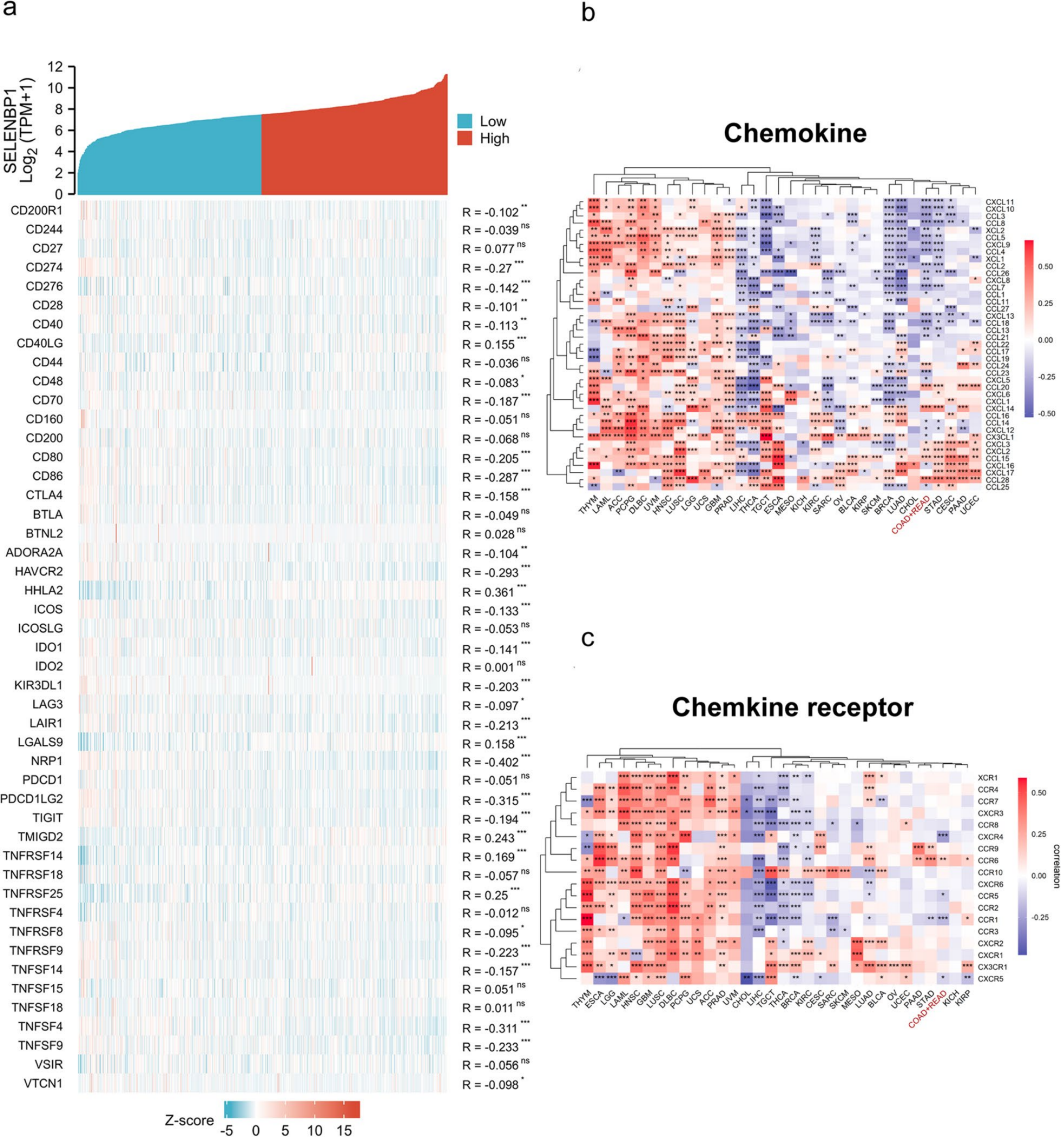
Correlation analysis between SELENBP1 gene expression and immune checkpoint markers, chemokines/chemokine receptors. **a** Correlation analysis of the level of SELENBP1 expression with several common immune checkpoint genes in CRC. **b–c** Correlation analysis of SELENBP1 with chemokines/receptors in CRC

Immune checkpoint inhibitors (ICIs) can eliminate tumors by suppressing the immune escape of tumor cells and enhancing the immune response of T cells [43]. Subsequently, we analyzed the correlation between the expression of SELENBP1 and common immune checkpoint-associated genes (Fig. 7a). We found that SELENBP1 expression is associated with multiple immune checkpoint markers, among which programmed cell death 1 ligand 1 (CD274/PD-L1) and TIM-3 have been used in clinical treatment [44]. Chemokines are able to influence tumorigenesis and development through immune pathways [45]. To elucidate the association between SELENBP1 expression and immune cell migration, we analyzed the association with chemokines/receptors (Fig. 7b–c). The results demonstrated that SELENBP1 expression was negatively associated with multiple immune cell-associated chemokines/receptors. Therefore, the low expression of SELENBP1 may contribute to the migration of immune cells in the tumor microenvironment.

### Correlation of SELENBP1 expression with chemotherapy and immunotherapy

**Fig. 8:**
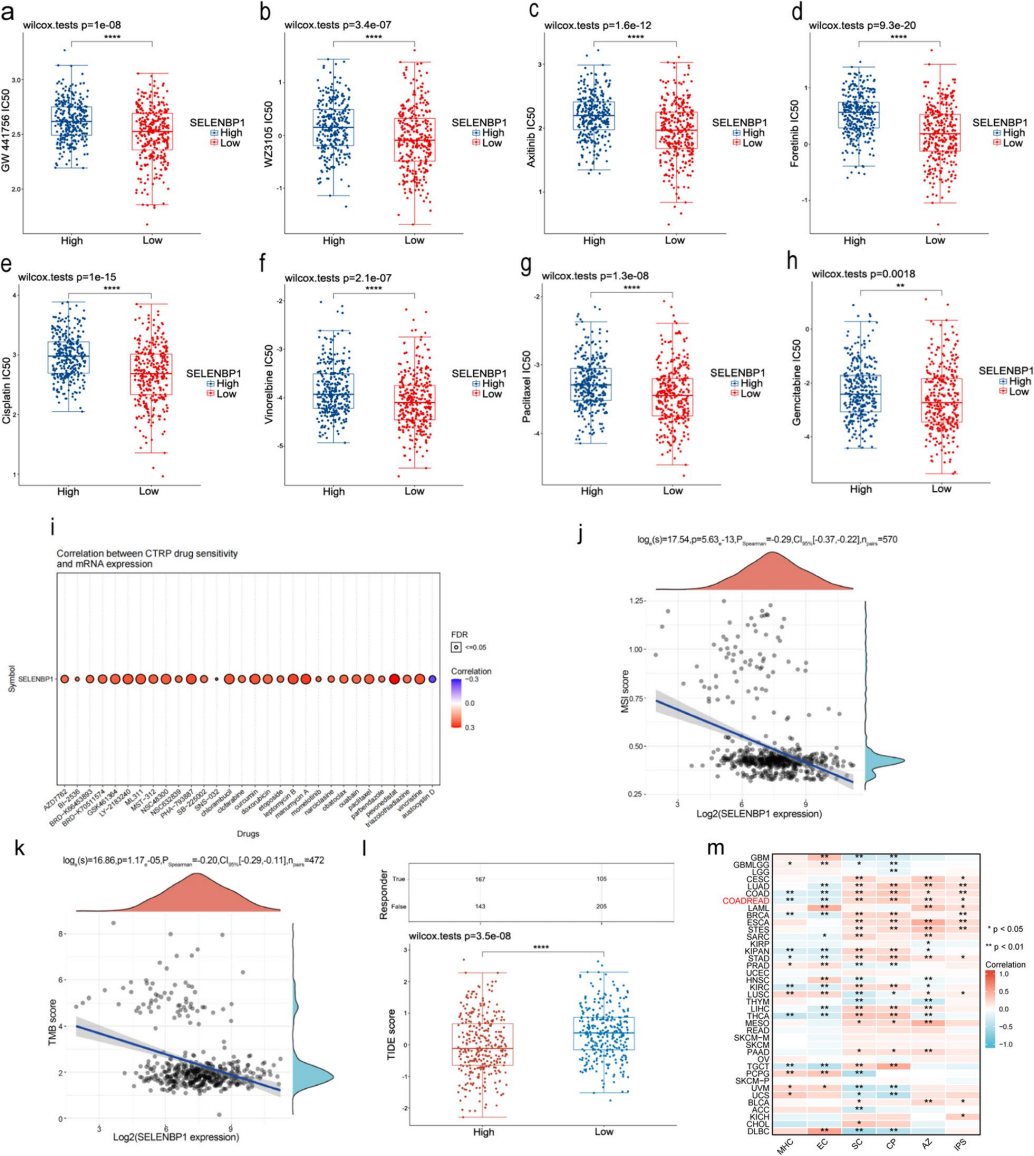
Prediction of drug sensitivity based on SELENBP1 expression in CRC. **a–h** IC50 of drugs in SELENBP1 high expression group versus low expression group. **i** Drugs screened based on the CTRP database that are highly sensitive to SELENBP1 mRNA expression. **j–k** Correlation analysis of SELENBP1 gene expression and TMB, MSI. **l** Relationship between SELENBP1 expression and the efficacy of ICB treatment. **m** Relationship between SELENBP1 expression and immunogenicity

To assess the effect of SELENBP1 expression on the efficacy of chemotherapy and immunotherapy, we calculated the drug half maximal inhibitory concentration (IC50) and TIDE values in the SELENBP1 high expression group versus the low expression group. First, we predicted the response of SELENBP1 expression to several common targeted and chemotherapeutic agents such as GW 441756, WZ3105, Axitinib, Foretinib, Cisplatin, Vinorelbine, Paclitaxel, Gemcitabine, based on GDSC database. The results showed that the IC50 values of all these drugs were higher in the high SELENBP1 expression group, which indicated better drug efficacy in the low SELENBP1 expression group (Fig. 8a–h). In addition, we also screened drugs sensitive to higher SELENBP1 mRNA expression based on the CTRP database. including etoposide, pevonedistat, vincristine clofarabine, chlorambucil, etc. (Fig. 8i). Then, we evaluated the correlation between SELENBP1 expression and TMB and MSI, and found a negative correlation between SELENBP1 and TMB and MSI in CRC (Fig. 8j–k). Finally, we also predicted SELENBP1 expression responsiveness to immune checkpoint inhibitors using TIDE algorithm based on expression profile data. The results revealed a high TIDE score in the SELENBP1 low expression group, suggesting a poor efficacy of SELENBP1 low expression in ICB (Fig. 8l). To verify that high SELENBP1 expression may be more effective for immunotherapy, we assessed the relationship between SELENBP1 expression and immunogenicity by immunophenotype score (IPS) analysis (Fig. 8m). The IPS score is mainly determined by the four main types of genes (e.g. activated CD4+ T cells, activated CD8+ T cells, effector memory CD4+ T cells, Treg, MDSCs): MHC molecules (MHC), immunomodulators (CP), effector cells (EC), and immunosuppressive cells (SC). Higher IPS scores were positively correlated with an increase in immunogenicity [46]. Based on our results, IPS scores increased with increasing SELENBP1 expression, and this result is consistent with the TIDE results, further confirming the effect of SELENBP1 on immunotherapy response.

## Discussion

CRC is one of the leading causes of cancer-related deaths. Screening and diagnosis of CRC is crucial for reducing incidence rate and mortality rate. Studies have shown that biomarkers can be used for early diagnosis, treatment options and prognostic assessment of CRC [47, 48]. However, there is still controversy regarding biomarkers for predicting the efficacy of CRC immunotherapy, therefore, it is an urgent issue to identify the best biomarkers to screen the CRC immunotherapy benefit population and predict the efficacy of immunotherapy.

Selenium as a microelement has important functions in physiological processes and cancer prevention [49]. Its role realized through the action on selenium containing proteins. SELENBP1 is a special selenium-containing protein, which may play an anti-cancer role in a variety of cancer types, and its expression level in cancer is lower than that in corresponding normal tissue [11]. The decreasing of SELENBP1 expression was related to the hypermethylation of SELENBP1. The methylation level of SELENBP1 promoter in CRC tissues was much higher than that in normal mucosa adjacent to cancer [50]. Available studies have shown that SELENBP1 expression correlates with disease-free survival (DFS) and OS in stage III CRC patients [14]. However, the specific biological function of SELENBP1 and the relationship with immune infiltration remain unclear.

Through a comprehensive analysis of multiple databases, we found that SELENBP1 was expressed at a low level in a variety of cancers. In addition, the expression of SELENBP1 was low in all subtypes of CRC. Our further analysis revealed that low expression of SELENBP1 usually indicates poor prognosis, such as shortened OS. To further investigate the functions of SELENBP1 in CRC, we performed enrichment analysis using TCGA data. The results showed that PD-1 signaling, signaling by interleukins, TCR signaling, collagen degradation, MHC class II antigen presentation, costimulation by the CD28 family, antigen processing cross presentation, was enriched to different degrees. SELENBP1 may influence the development of CRC by affecting these pathways, leading to poor prognosis for CRC patients.

In the past few years, immunotherapy has developed rapidly and has become an important tool in the treatment of CRC [51]. Our study found that SELENBP1 expression was negatively correlated with multiple immune cells and that SELENBP1 may affect the prognosis of CRC patients by influencing the degree of immune infiltration. With SELENBP1 expression, Stromal Score, Immune Score, and ESTIMATE Score of CRC decreased significantly. In patients with high degree of tumor immune infiltration, OS, RFS were significantly prolonged. In addition, our study showed that SELENBP1 is closely related to immune-related chemokines/receptors. Several chemokines such as CCL2/3/4, CXCL9/10, CXCR4 have been shown to play important roles in the immune infiltration of CRC [52–54].

Through the analysis we found negative correlation between SELENBP1 expression and TMB and MSI, however, based on TMB, MSI did not accurately predict the efficacy of ICB therapy. The effectiveness of ICB treatment is influenced by various factors such as PD-L1 expression [55], MSI [56], interferon signaling [57], intestinal microbiota [58], cytotoxic T cell infiltration [59], and TMB [60]. In this regard, we analyzed the effectiveness of SELENBP1 expression and immunotherapy using the TIDE integrated algorithm, and the results showed that high SELENBP1 expression is more sensitive to immunotherapy.

In summary, this analysis showed that SELENBP1 may be lower expressed in CRC due to mutation and DNA methylation and is associated with poorer survival. Reduced SELENBP1 expression increased the invasiveness of CRC. In addition, SELENBP1 expression was also associated with immune infiltration and the efficacy of immunotherapy. Therefore, SELENBP1 is an important prognostic biomarker in CRC, and further exploration of the relationship between SELENBP1 and immune infiltration is essential to elucidate the value of SELENBP1 in the treatment of CRC.

## Supplementary Information

**Additional file 1**. **Supplementary Figs. 1–4. Supplementary Fig. 1. **The scans of immunoblots from Fig. 2a. The line indicated the band position for each protein. M, molecular weight markers; N, normal tissue; C, colorectal cancer tissue. All tissues were derived from human samples. **Supplementary Fig. 2.** Relationship between SELENBP1 expression and prognosis of cancer patients. (a–d, i–k) OS was significantly prolonged in patients with high SELENBP1 expression in HNSC, LUAD, LIHC, THCA, MESO, SARC, BLCA. (e–h, l–m) Reliability of SELENBP1 expression in HNSC, LUAD, LIHC, THCA, MESO, SARC, BLCA in predicting OS. **Supplementary Fig. 3.** Correlation of SELENBP1 with immune cell infiltration in CRC. (a) Lollipop plot showing the correlation of SELENBP1 with 24 immune cell subsets in CRC. (b–r) Scatter plots showing positive correlation of SELENBP1 with eosinophils, B cells and Th17 cells, and negative correlation with macrophages, Th1 cells, neutrophils, Th2 cells, Tgd, NK cells, T helper cells, Tem, cytotoxic cells, Tcm, CD8 T cells, aDC and DC. **Supplementary Fig. 4.** Immune cell subgroup-based analysis of SELENBP1 expression levels in relation to OS in CRC patients(a)  Decreased B cells, (b) Enriched B cells, (c) Enriched CD4+T cells, (d)Enriched CD8+T cells, (e) Decreased CD8+T cells, (f)Enriched eosinophils, (g)Decreased eosinophils, (h)Enriched macrophages, (i) Decreased macrophages, (j)Enriched mesenchymal stem cells, (k) Decreased mesenchymal stem cells, (l)Enriched Natural killer T cells, (m) Decreased Natural killer T cells, (n) Enriched regulatory T cells, (o) Decreased regulatory T cells, (p) Enriched type 1 T-helper cells, (q) Decreased type 1 T-helper cells, (r) Enriched type 2 T-helper cells.

## Data Availability

The datasets used and analyzed during the current study are available from the publicly open databases of TCGA (http://cancergenome.nih.gov/), GTEx (https://cancergenome.nih.gov/), Oncomine (https://www.oncomine.org/resource/main.html), UALCAN ( http://ualcan.path.uab.edu/index.html), CancerSEA (http://biocc.hrbmu.edu.cn/CancerSEA/), cBioPortal (http://www.cbioportal.org/index.do), Kaplan⁃Meier Plotter ( http://kmplot.com/analysis/), GDSC (https://www.cancerrxgene.org/), and CTRP (http://www.broadinstitute.org/ctrp/).

## Abbreviations

ACC: Adrenocortical carcinoma
aDCs: Activated dendritic cells
BLCA: Bladder urothelial carcinoma
BRCA: Breast invasive carcinoma
CCL2/3/4: C–C Motif Chemokine Ligand 2/3/4
CD274/PD-L1: Programmed cell death 1 ligand 1
CESC: Cervical squamous cell carcinoma and endocervical adenocarcinoma
CHOL: Cholangiocarcinoma
COAD: Colon adenocarcinoma
CRC: Colorectal cancer
CTRP: The Cancer Therapeutics Response Portal
CXCL9/10: C-X-C motif ligand 9/10
CXCR: C-X-C chemokine receptor
CXCR4: C-X-C chemokine receptor 4
DC: Dendritic cell
DEG: Differentially expressed genes
DFS: Disease-free survival
DLBC: Lymphoid neoplasm diffuse large B-cell lymphoma
ECL: Enhanced chemiluminescence assay
ESCA: Esophageal carcinoma
FDR: False discovery rate
GBM: Glioblastoma multiforme
GDSC: Genomics of Drug Sensitivity in Cancer
GO/KEGG: Gene ontology/Kyoto encyclopedia of genes and genomes
GSEA: Gene Set Enrichment Analysis
GTEx: Genotype-tissue expression
HNSC: Head and neck squamous cell carcinoma
ICB: Immune checkpoint blockade
ICGC: International Cancer Genome Consortium
ICI: Immune Checkpoint Inhibitors
IC50: Half maximal inhibitory concentration
IHC: Immunohistochemistry
IL-17: Interleukin-17
KICH: Kidney chromophobe
KIRC: Kidney renal clear cell carcinoma
KIRP: Kidney renal papillary cell carcinoma
KM: Kaplan–Meier
LAML: Acute myeloid leukemia
LGG: Brain lower grade glioma
LIHC: Liver hepatocellular carcinoma
LUAD: Lung adenocarcinoma
LUSC: Lung squamous cell carcinoma
MESO: MesotheliomaMHC class II
MSI: Microsatellite instability
NES: Normalized enrichment score
NK: Natural killer cell
OS: Overall survival
OV: Ovarian serous cystadenocarcinoma
PAAD: Pancreatic adenocarcinoma
PCPG: Pheochromocytoma and Paraganglioma
PD-1: Programmed death 1
PRAD: Prostate adenocarcinoma
READ: Rectum adenocarcinoma
RFS: Recurrence-free survival
ROC: Receiver Operating Characteristic
SARC: Sarcoma
SELENBP1: Selenium-binding protein 1
SKCM: Skin Cutaneous Melanoma
STAD: Stomach adenocarcinoma
TCGA: The cancer genome atlas
Tcm: T central memory
TCR: T-cell-receptor
Tem: T effector memory
TGCT: Testicular Germ Cell Tumors
THCA: Thyroid carcinoma
THYM: Thymoma
Th1: T helper 1
Th2: T helper 2
Th17: T helper 17
TIDE: Tumor immune dysfunction and rejection
TIM-3: T cell immunoglobulin domain and mucin domain-3
TPM: Transcripts per kilobase of exon model per million mapped reads
TMB: Tumor mutation burden
UCEC: Uterine corpus endometrial carcinoma
WB: Western blotting
