# Supplementary material for: Tumor microenvironment-related gene selenium-binding protein 1 (SELENBP1) is associated with immunotherapy efficacy and survival in colorectal cancer
Source: BMC Gastroenterol. 2022 Oct 17;22:437. doi: 10.1186/s12876-022-02532-2 (PMC9575293; doi:10.1186/s12876-022-02532-2)
Supplement: Supplementary file 1 — Additional file 1. Supplementary Figs. 1–4. Supplementary Fig. 1. The scans of immunoblots from Fig. 2a. The line indicated the band position for each protein. M, molecular weight markers; N, normal tissue; C, colorectal cancer tissue. All tissues were derived from human samples. Supplementary Fig. 2. Relationship between SELENBP1 expression and prognosis of cancer patients. (a–d, i–k) OS was significantly prolonged in patients with high SELENBP1 expression in HNSC, LUAD, LIHC, THCA, MESO, SARC, BLCA. (e–h, l–m) Reliability of SELENBP1 expression in HNSC, LUAD, LIHC, THCA, MESO, SARC, BLCA in predicting OS. Supplementary Fig. 3. Correlation of SELENBP1 with immune cell infiltration in CRC. (a) Lollipop plot showing the correlation of SELENBP1 with 24 immune cell subsets in CRC. (b–r) Scatter plots showing positive correlation of SELENBP1 with eosinophils, B cells and Th17 cells, and negative correlation with macrophages, Th1 cells, neutrophils, Th2 cells, Tgd, NK cells, T helper cells, Tem, cytotoxic cells, Tcm, CD8 T cells, aDC and DC. Supplementary Fig. 4. Immune cell subgroup-based analysis of SELENBP1 expression levels in relation to OS in CRC patients(a) Decreased B cells, (b) Enriched B cells, (c) Enriched CD4+T cells, (d)Enriched CD8+T cells, (e) Decreased CD8+T cells, (f)Enriched eosinophils, (g)Decreased eosinophils, (h)Enriched macrophages, (i) Decreased macrophages, (j)Enriched mesenchymal stem cells, (k) Decreased mesenchymal stem cells, (l)Enriched Natural killer T cells, (m) Decreased Natural killer T cells, (n) Enriched regulatory T cells, (o) Decreased regulatory T cells, (p) Enriched type 1 T-helper cells, (q) Decreased type 1 T-helper cells, (r) Enriched type 2 T-helper cells. [file 12876_2022_2532_MOESM1_ESM.docx]

**
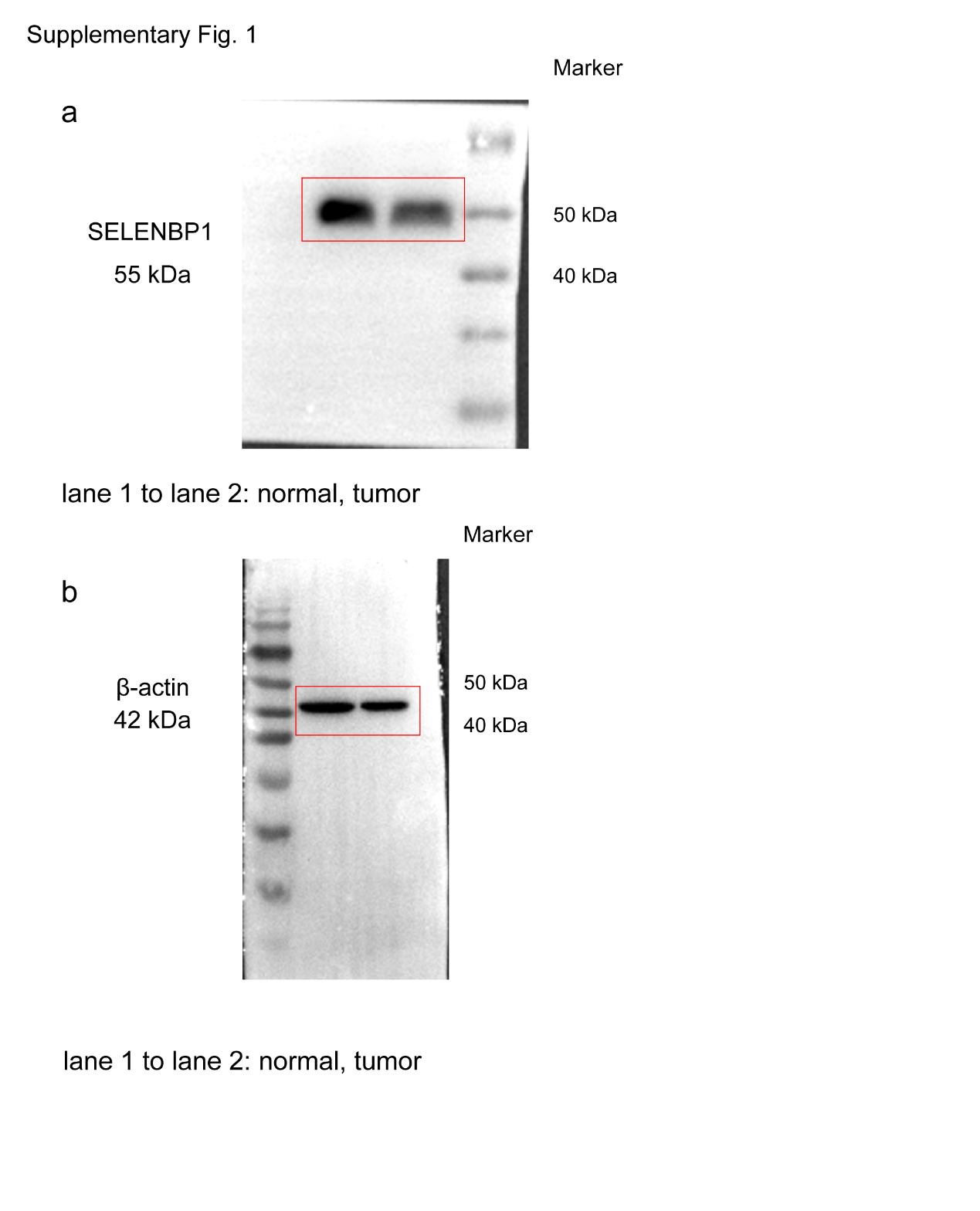
Supplementary Fig. 1. The scans of immunoblots from Fig. 2a.**

The line indicated the band position for each protein. M, molecular weight markers; N, normal tissue; C, colorectal cancer tissue. All tissues were derived from human samples.


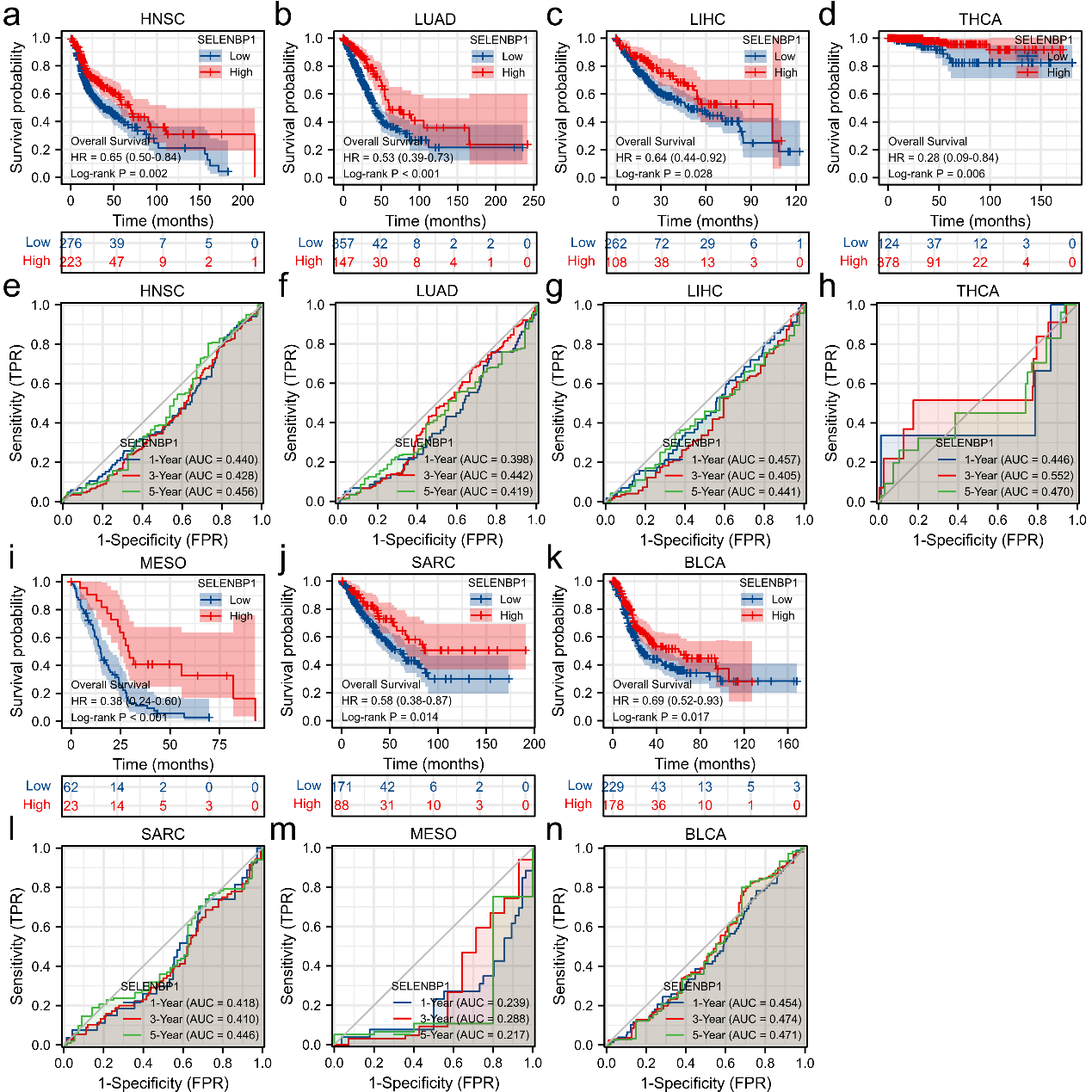


**Supplementary Fig. 2**. **Relationship between SELENBP1 expression and prognosis of cancer patients.**

**(a-d, i-k)** OS was significantly prolonged in patients with high SELENBP1 expression in HNSC, LUAD, LIHC, THCA, MESO, SARC, BLCA. **(e-h, l-m)** Reliability of SELENBP1 expression in HNSC, LUAD, LIHC, THCA, MESO, SARC, BLCA in predicting OS.


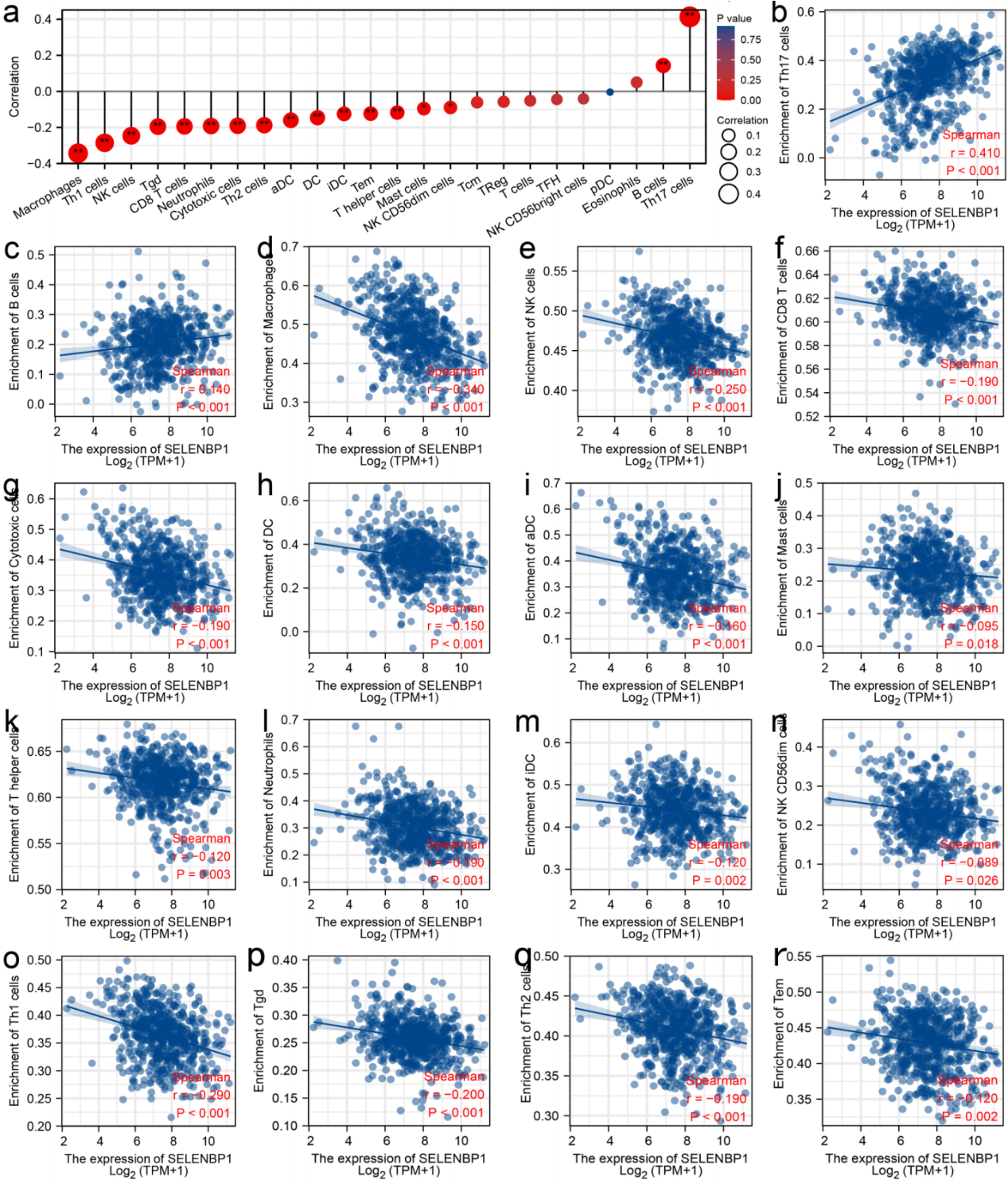


**Supplementary Fig. 3**. **Correlation of SELENBP1 with immune cell infiltration in CRC.**

**(a**) Lollipop plot showing the correlation of SELENBP1 with 24 immune cell subsets in CRC.**(b-r)** Scatter plots showing positive correlation of SELENBP1 with eosinophils, B cells and Th17 cells, and negative correlation with macrophages, Th1 cells, neutrophils, Th2 cells, Tgd, NK cells, T helper cells, Tem, cytotoxic cells, Tcm, CD8 T cells, aDC and DC.


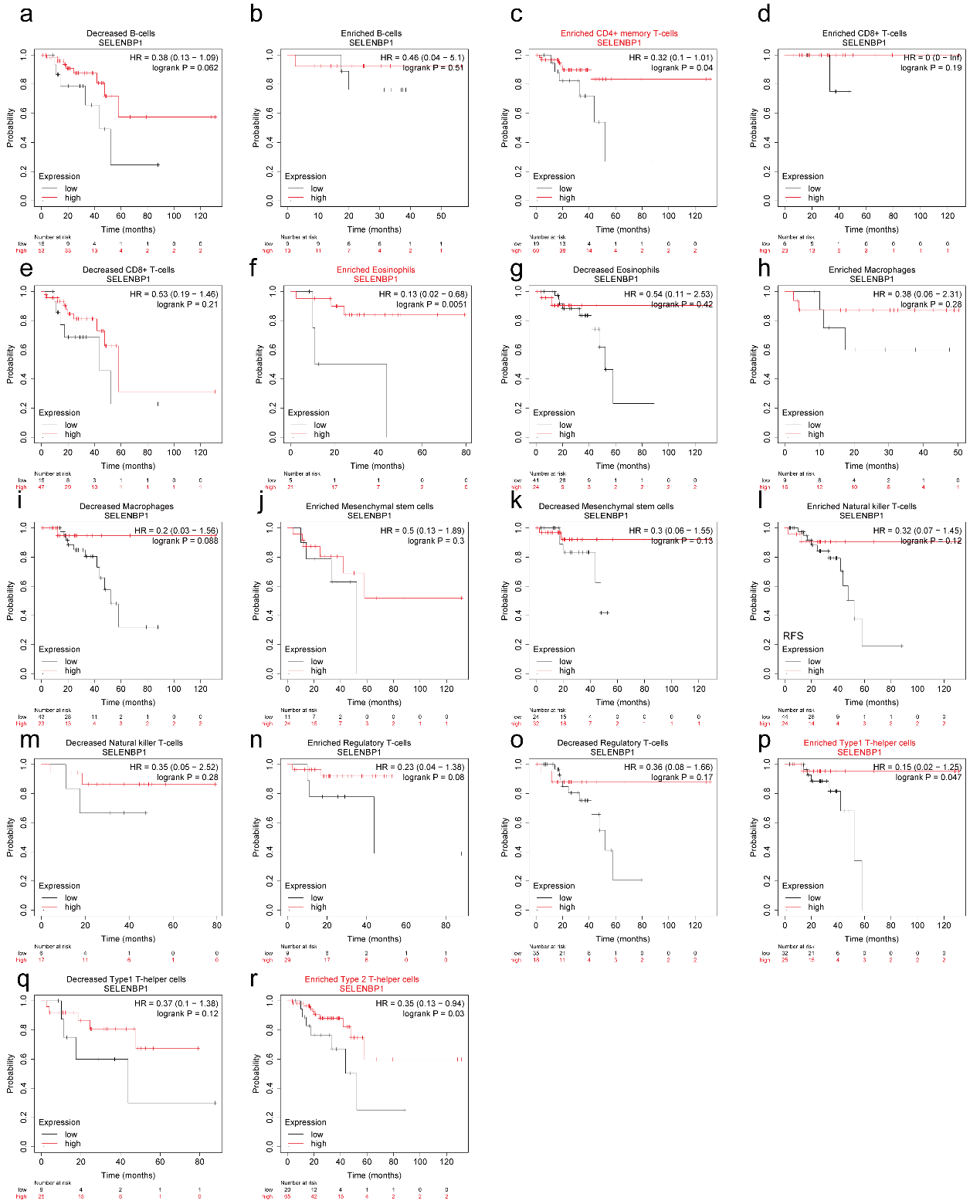


**Supplementary Fig. 4. Immune cell subgroup-based analysis of SELENBP1 expression levels in relation to OS in CRC patients**

1. Decreased B cells, **(b)**Enriched B cells, **(c)**Enriched CD4+T cells, **(d)**Enriched CD8+T cells, **(e)**Decreased CD8+T cells, **(f)**Enriched eosinophils, **(g)**Decreased eosinophils, **(h)**Enriched macrophages, **(i)**Decreased macrophages, **(j)**Enriched mesenchymal stem cells, **(k)**Decreased mesenchymal stem cells, **(l)**Enriched Natural killer T cells, **(m)**Decreased Natural killer T cells, **(n)**Enriched regulatory T cells, **(o)**Decreased regulatory T cells, **(p)**Enriched type 1 T-helper cells, **(q)**Decreased type 1 T-helper cells, **(r)**Enriched type 2 T-helper cells.
